# Impact of a tuberculosis treatment adherence intervention versus usual care on treatment completion rates: results of a pragmatic cluster randomized controlled trial

**DOI:** 10.1186/s13012-020-01067-y

**Published:** 2020-12-11

**Authors:** Lisa M. Puchalski Ritchie, Monique van Lettow, Austine Makwakwa, Ester C. Kip, Sharon E. Straus, Harry Kawonga, Jemila S. Hamid, Gerald Lebovic, Kevin E. Thorpe, Merrick Zwarenstein, Michael J. Schull, Adrienne K. Chan, Alexandra Martiniuk, Vanessa van Schoor

**Affiliations:** 1 Department of Medicine, University of Toronto, 6 Queen’s Park Crescent West, Third Floor, Toronto, ON M5S 3H2 Canada; 2 Li Ka Shing Knowledge Institute, St. Michaels Hospital, St. Michael’s Hospital, 30 Bond St, Toronto, ON M5B 1W8 Canada; 3 Department of Emergency Medicine, University Health Network, Toronto General Hospital, 200 Elizabeth Street, RFE G-480, Toronto, M5G 2C4 Canada; 4 Institute of Health Policy, Management and Evaluation, University of Toronto, 155 College Street, Toronto, M5T 3M7 Canada; 5 Dignitas International, Zomba, Malawi; 6 Dalla Lana School of Public Health, University of Toronto, 155 College Street, Toronto, M5T 3M7 Canada; 7 National TB Program, Ministry of Health, Lilongwe, Malawi; 8 School of Epidemiology and Public Health, University of Ottawa, Room 101, 600 Peter Morand Crescent, Ottawa, ON I1G 5Z3 Canada; 9 Applied Health Research Centre, Li Ka Shing Knowledge Institute, St. Michael’s Hospital, 30 Bond St, Toronto, ON M5B 1W8 Canada; 10 Department of Family Medicine, Western University, London, ON Canada; 11 Department of Family Medicine, Schulich School of Medicine & Dentistry, Western University, 1151 Richmond St, London, ON N6A 5C1 Canada; 12 Division of Infectious Diseases, Department of Medicine, Sunnybrook Health Sciences Center, University of Toronto, c/o H2-66, 2075 Bayview Avenue, Toronto, ON M4N 3M5 Canada; 13 Dignitas International Toronto, C/O ICES attention Michael Schull, 2075 Bayview Avenue, G106, Toronto, ON M4N 3M5 Canada; 14 George Institute for Global Health, Sydney, Australia; 15 The University of Sydney, Edward Ford Building, Sydney, NSW Australia

**Keywords:** Lay health workers, Community health workers, Educational outreach, Reminders, Peer support network, Tuberculosis, Cluster randomized trial

## Abstract

**Background:**

With the global shortage of skilled health workers estimated at 7.2 million, outpatient tuberculosis (TB) care is commonly task-shifted to lay health workers (LHWs) in many low- and middle-income countries where the shortages are greatest. While shown to improve access to care and some health outcomes including TB treatment outcomes, lack of training and supervision limit the effectiveness of LHW programs. Our objective was to refine and evaluate an intervention designed to address common causes of non-adherence to TB treatment and LHW knowledge and skills training needs.

**Methods:**

We employed a pragmatic cluster randomized controlled trial. Participants included 103 health centres (HCs) providing TB care in four districts in Malawi, randomized 1:1 stratified by district and HC funding (Ministry of Health, non-Ministry funded). At intervention HCs, a TB treatment adherence intervention was implemented using educational outreach, a point-of-care reminder tool, and a peer support network. Clusters in the control arm provided usual care. The primary outcome was the proportion of patients with successful TB treatment (i.e., cure or treatment completion). We used a generalized linear mixed model, with district as a fixed effect and HC as a random effect, to compare proportions of patients with treatment success, among the trial arms, with adjustment for baseline differences.

**Results:**

We randomized 51 HCs to the intervention group and 52 HCs to the control group. Four intervention and six control HCs accrued no eligible patients, and 371 of 1169 patients had missing outcome, HC, or demographic data, which left 74 HCs and 798 patients for analysis. Randomization group was not related to missing outcome, however, district, age, and TB type were significantly related and included in the primary analysis model. Among the 1153 patients with HC and demographic data, 297/605 (49%) and 348/548 (64%) in the intervention and control arms, respectively, had treatment success. The intervention had no significant effect on treatment success (adjusted odds ratio 1.35 [95% confidence interval 0.93–1.98]).

**Conclusion:**

We found no significant effect of the intervention on TB treatment outcomes with high variability in implementation quality, highlighting important challenges to both scale-up and sustainability.

**Trial registration:**

ClinicalTrials.gov NCT02533089. Registered August 20, 2015.

**Supplementary Information:**

The online version contains supplementary material available at 10.1186/s13012-020-01067-y.

Contributions to the literature

- This is one of few studies to date that has evaluated an intervention designed to address LHW training needs, with the goal of improving the TB care provided by LHWs and through this improve TB outcomes.
- We identified important barriers to implementation quality, scalability, and sustainability in the use of an educational outreach approach to address LHW training needs, and we highlight challenges to the use of routine data for monitoring and evaluation of implementation programs.
- Lessons learned provide information that will be important to future intervention development and implementation planning in Malawi and that may also be of benefit for implementation planning in other settings where TB care is provided mainly by LHWs.

## Background

Tuberculosis (TB) remains an important cause of morbidity and mortality globally, with an estimated 10 million new TB notifications and more than 1.2 million TB deaths in 2018 [1]. Although there have been improvements in treatment success rates [2], incomplete treatment continues to contribute to the high TB burden. Continued efforts to improve treatment success are needed.

With the global shortage of skilled health workers currently estimated at 7.2 million and rising [3], outpatient TB care is commonly task-shifted to lay health workers (LHWs) in many low- and middle-income countries (LMICs). LHWs therefore play a critical role in addressing the high TB burden in such settings.

Evidence from systematic reviews has shown that LHWs have generally small but positive effects on TB treatment completion rates [4, 5]. However, lack of training and supervision are recognized as important barriers to optimal functioning of LHW programs [6, 7]. Given the importance and increasing role of LHWs in providing TB-related and other essential health care in many LMICs, low-cost, effective options to improve LHW training and supervision are needed.

Malawi is among the countries hardest hit by the global shortage of skilled health workers: with 0.16 doctors and 2.53 nurses and midwives per 10,000 population in 2016, and the numbers steadily declining over the past decade [8, 9]. Despite having one of the largest national LHW workforces, estimated at 12,000 in 2015 [10], this number is inadequate to achieve the policy target of one LHW per 1000 population [11].

In Malawi, LHWs providing TB care are part of a cadre of paid health workers who provide a link between communities and the health system; they perform various health promotion and prevention tasks, as well as a limited number of curative tasks, including outpatient TB treatment [12]. Despite substantial improvements, TB treatment success rates remain below the 95% target set out in the country’s 2012–2016 strategic plan [13]. As the primary providers of TB care, LHWs will play a pivotal role in achieving this target and in reducing rates of TB incidence, morbidity, and mortality.

Qualitative formative research conducted by our team revealed training needs among LHWs providing TB care in Malawi [14]. Specifically, LHWs identified their own lack of knowledge (concerning the disease and its treatment) and skills (related to patient-provider interactions and treatment documentation) as the primary barriers to their work. From these findings, we developed a TB treatment adherence intervention to address training needs, in terms of both knowledge and skills, and employed on-site, peer-led educational outreach, and a point-of-care reminder tool to support implementation. We pilot tested the intervention in a cluster randomized trial in a single district of Malawi [15, 16], which demonstrated the feasibility and acceptability of the intervention and implementation strategy employed. The results, though not statistically significant, showed some improvement in treatment success, suggesting that a full-scale trial would be both feasible and useful.

We sought to refine the intervention and implementation strategy on the basis of feedback from participants and our experience during the pilot study, and also to evaluate the effectiveness of the refined intervention and implementation strategy on a larger scale. We also wanted to understand the barriers and facilitators to institutionalization of the intervention in Malawi, especially its scalability and sustainability, as well as to understand potential use of the implementation strategy to address LHW training and supervision needs in other non-TB areas of care.

## Study aim

Our objectives were to refine a previously piloted TB treatment adherence intervention, designed to give LHWs the knowledge and skills needed to address common causes of TB treatment non-adherence [15, 16], and to evaluate its effectiveness in improving TB treatment outcomes.

## Methods

### Trial design

The complete study protocol was previously published [17]. In brief, we used a mixed methods design, consisting of a pragmatic cluster randomized controlled trial and a process evaluation, to evaluate the effectiveness of the intervention on patients’ TB treatment outcomes and to understand barriers and facilitators to implementation, scalability, and sustainability of the intervention. In Malawi, patients commonly receive outpatient TB care from several LHWs; therefore, a cluster trial with allocation at the HC level was chosen, to prevent contamination. Our mixed methods design was informed by the RE-AIM framework [18], with results of the trial reported here and detailed findings of the process evaluation reported separately.

### Setting and participants

The study was conducted in four districts in the South East zone of Malawi. Of the 109 health centres (HCs) assessed for eligibility, 103 routinely provided TB care, were expected to remain open for the study duration, and were therefore eligible for inclusion. All LHWs routinely providing TB care at intervention sites were eligible and invited to participate; refusal to participate was the only exclusion criterion.

### Randomization

The HCs were randomized, at a 1:1 ratio, stratified by district and funding source (Ministry of Health or non-Ministry funding). Each district health office provided a list of HCs providing TB care and their funding source. A computer-generated random number list, stratified by district and funding source, was created centrally by a study team member not involved in the trial. The study coordinator used the computer-generated list to allocate HCs to the intervention or control group, with all HCs allocated at one time. Once allocation was complete, letters were sent to intervention HCs, briefly describing the study and asking that TB-focus LHWs be sent for training as peer trainers (PT). HCs in the control arm received no communication and provided usual care. Individual patients were not enrolled in the trial; rather, outcomes were obtained from TB registers, which include all patients registered for TB care in the district.

As training is a routine expectation of HC staff, LHWs were invited but not required to participate in the intervention and participation in training was approved by both the national and district TB offices; individual consent was not required.

### Intervention

A detailed description of the piloted TB treatment adherence intervention was previously published [15, 16]. In brief, our implementation strategy employed peer-led educational outreach and a point-of-care tool to implement a TB treatment adherence intervention designed to address LHW training needs and common barriers to TB treatment adherence, by improving LHW knowledge and patient counselling skills. Selection of implementation strategies was based on mapping of evidence-based approaches to addressing barriers and facilitators to the provision of evidence-based TB care by LHWs, as identified through formative work [14, 15]. Selection and tailoring of implementation strategies was further informed by considerations of feasibility, scalability, and sustainability in the Malawi health care context.

In the current study, a medication dosing table and a peer support network were added to our original implementation strategy based on feedback from PTs and LHWs in the pilot study. Drafts of the revised point-of-care tool were usability tested and revised iteratively by LHW participants from the pilot study, who represented an experienced group, and by a novice group from the original study district who were not part of the current study and who did not participate in the pilot study. Usability testing involved both concurrent and retrospective think-aloud approaches [19]. Participants were first asked to think out loud while using the point-of-care tool in mock patient interactions (concurrent component); two study team members (LMPR, ECK) recorded their observations of these interactions, without probing. Following mock patient interactions (typically three), participants provided feedback on usability of the tool and suggestions for improvement (retrospective component). Revisions and testing continued until no further usability issues were identified.

Changes to the point-of-care tool included minor revisions to pictorials to show the patient-LHW interaction (opportunity to discuss with patients the importance of communicating questions or concerns to the care team) and addition of the medication dosing table for easy reference during patient encounters. Changes to the educational outreach included addition of a supportive supervision component to the PT training and lengthening of the period for cascade training. A peer support network (to facilitate communication outside quarterly PT meetings with the study team) was facilitated by sharing cell phone numbers among PTs within each district and providing a small quarterly stipend (approximately 1 USD) for phone credit. No other encouragement or support for the peer support network was provided.

### Intervention implementation

A detailed description of the TB treatment adherence intervention and implementation strategy, which follows the template for intervention description and replication (TIDieR) format [20], appears in Table 1 (see TIDieR checklist, Additional file 1). The letters sent to HCs provided details about location, time, and purpose of the PT training. Only one TB-focus LHW per site received PT training. Travel, meal, and accommodation expenses were reimbursed; stipends for attendance were not provided. LHWs from two small adjacent districts were trained together. PT training was provided over 1 week by LMPR, in English, with support from a socio-linguistic level translator, with an additional study team member (HK) present to support training in one large district. LMPR is an experienced trainer who also led the PT training in the pilot study. Training involved a combination of didactic lectures, interactive discussions, and role playing to allow for practice and supportive feedback. The final day of training included an interactive discussion of potential approaches to the cascade training, including options for addressing anticipated challenges to training, particularly lack of training stipends. The importance of not sharing study materials or teachings with peers from non-intervention sites was also discussed.

**Table 1:** Description of intervention

Title	TB adherence intervention
Rationale/goals	Goal of the intervention is to improve TB care provided by LHWS and in particular treatment adherence counselling and support to address factors related to incomplete treatment, and through this improve successful treatment rates and patient outcomes.
Materials and procedures	The TB adherence intervention required providers to assess adherence, to provide education and counselling based on risk factors for non-adherence, and to address any patient questions or concerns at each clinical encounter. Three implementation strategies were employed to support implementation: on-site peer-led educational outreach, point-of-care reminder tool, and peer support network. Educational outreach employed both didactic and interactive techniques including case-based discussions and role playing to convey TB-specific knowledge and job-specific skills and to allow for practice and sharing of ideas and experiences between LHWs. Topics included TB transmission, treatment, and consequences of poor adherence; the interaction of TB and HIV; common barriers to adherence; patient-provider communication skills including approaches to preventing and addressing non-adherence; and methods and benefits of supportive supervision. Peer trainers were trained both in the content and approach to teaching off-site by a master trainer (LMPR) and provided with a training manual and resources (flip chart, markers, etc.) and received certificates at completion of training. Peer trainers led educational outreach sessions at their base health centre during regular work hours. Peer trainers were asked to provide eight sessions, each a minimum of 60 min in duration, over a 4-month period, and to provide supportive supervision throughout the study period. Point-of-care tool was designed as an A4 size desktop flip chart that can be folded to be carried for field visits. The patient side of the tool uses simple pictorials to illustrate key messages, for use in patient education and adherence counselling. The provider side of the tool provides a guide to discussing adherence and providing adherence counselling, as well as clinical support for management of side effects. A third page included the basic TB treatment dosing regimens for easy reference during patient encounters. The tool was revised based on feedback from LHW participants in the pilot study and further revised through usability testing with LHWs in the pilot district (not part of the current study) prior to implementation. Peer support network. A small (approximately one USD) amount of money was provided quarterly for phone credit to facilitate development of a peer-support- network among peer trainers, who were trained together but are generally widely dispersed across large geographical areas. Networking was further supported by quarterly in-person meetings with peer trainers and the study team.
Intervention provider	TB-focus LHWs from each intervention site were trained as peer trainers. TB focus LHWs are general LHWs with varying years of LHW and TB experience, who receive an additional 2 weeks of TB-specific training from the ministry of health and are responsible for outpatient TB care at the health centre level. Note, at least one TB focus LHW had not received their TB focus training at the start of the intervention, but did receive it shortly after they received the intervention training.
Method of delivery	Educational outreach sessions were provided face to face.
Location/context	Sessions took place at the LHWs base health centre during regular work hours, typically afternoons on less busy days of the week (i.e., mid-week).
Dose	Peer trainers were to provide eight sessions, each lasting a minimum of 60 min, over a 4-month period, and to provide supportive supervision throughout the study period.
Tailoring	Additional sessions as make-ups for staff that missed sessions, joined the health centre team outside the initial training period or for LHWs who initially declined to participate but later requested training, were left to the discretion of the peer trainers. Several suggested approaches to supportive supervision were discussed and practiced during peer trainer training, with the form used left to the discretion of individual peer trainers.
Modifications	Training period extended formally from 2 to 3 weeks depending on the timing of the peer trainer training in the district to accommodate staff absences due to annual leave/illness/TB focus training/national exams and delay in dissemination of training manuals. In additional to individual make-up sessions as outlined and planned for through tailoring, some peer trainers trained a second cohort later in the course of the study, due to staffing changes and/or to train LHWs who had initially declined to participate in training.
Fidelity	Fidelity information was collected informally during quarterly peer-trainer meetings, field visits, and interviews in two companion qualitative studies. High variability in the proportion of LHWs regularly providing TB care who participated in the training was reported, varying from zero to all LHWS at a given health centre trained. Interviews also revealed some variability in the number and duration of sessions provided by peer trainers, with some combining sessions into fewer longer sessions.

The PTS were given training materials, including manuals in Chichewa, stationary, point-of-care tools, and log books for recording training details, and received certificates from the study team upon completing the training. PTs were asked to provide cascade training onsite at their respective base HCs during regular work hours. LHWs routinely providing TB care were to be invited but not required to attend this training. PTs were asked to provide 8 sessions (minimum 60 min each) over 4 months. The training period was extended by 2 to 3 weeks because of delays in delivery of training manuals and to address absences of PTs and/or LHW trainees due to annual leave and/or other off-site training. Quarterly meetings of PTs with the study team provided opportunities to raise questions or concerns and to share experiences of the initial cascade training and ongoing implementation of the intervention. Quarterly phone credit was provided to allow for peer-to-peer support between the quarterly meetings. Additional support was available through phone contact with the study coordinator as needed and in-person check-ins from Dignitas mentors during routine site visits.

Dignitas International was an academic non-governmental organization (NGO) providing support and mentorship to front-line clinical staff and conducting research in the study districts. Dignitas mentors were clinical staff based in each participating district. As a result of restructuring of catchment areas for NGOs in the area, Dignitas International was withdrawn from two of the four study districts approximately 4 months after the cascade training began, after which Dignitas mentors were no longer available to support PTs in these districts.

### Control arm

No intervention was implemented in the control HCs. LHWs receive general pre-service training, which includes a brief overview of TB surveillance, diagnosis, and treatment and the role of LHWs in TB care. TB-specific training beyond the pre-service period is generally left to the discretion of the TB-focus LHW at the HC level. In addition, although TB care is provided free of charge at all HCs, according to national guidelines, operationalization and supervision of TB services at individual HCs is at the discretion of the local TB-focus LHW(s) with substantial variability in approach and level of supervision provided. No specific reference materials, other than the national TB guideline, are routinely available; in particular, the point-of-care tool developed as part of the implementation strategy was not available to LHWs at control sites.

### Data collection and outcome measures

Outcome data were obtained from Ministry of Health records. TB registers are paper-based logs maintained at registration HCs and include basic patient demographic data, name of the treating HC, patient’s HIV status, and details of TB diagnosis and treatment. Treatment records maintained at the treating HCs are submitted to the pertinent registration centre at the end of treatment, when treatment outcomes are entered into the register. TB registers in each of the four participating districts were digitized by trained research assistants, double-entered by trained data entry clerks, and verified by a data manager. Data were abstracted for all patients who started treatment on or after October 1, 2016, and completed treatment or otherwise exited the study on or before September 30, 2017.

Despite ethics and Ministry approval for digitization of the TB registers, one site prohibited digitization of identifying data, which left a small number of cases that could not be checked for transfers from other participating districts. Two patients initially defaulted and then restarted their treatment within the study period; final outcomes for the second course of treatment were used in the analysis. No instances of patient transfer from one study district to another study were identified, with final outcomes therefore maintained as transferred out.

Outcomes in the TB registers were classified according to World Health Organization definitions [21]. The primary outcome was the proportion of patients with successful treatment, defined as the combined total of patients with treatment outcomes of “cure” and “treatment complete.” Additional outcomes of interest were the secondary trial outcome of proportion of default cases (treatment interrupted for at least two consecutive months) and the subgroup analysis of proportion of successes among patients with HIV co-infection (pre-specified in the trial registration). Covariates of interest were age, sex, TB type, HIV status, HC, and district. All covariates of interest were pre-specified in the ethics submission to encompass factors related to TB treatment outcomes in the pilot study and/or the adherence literature, factors related to our study design, and data routinely collected and included in the TB register. See Table 2 for the list of variables and their definitions.

**Table 2:** Variable definitions

Variable	Definition
TB type	**Pulmonary TB** refers to any confirmed or clinically diagnosed case of TB involving the lung parenchyma or the tracheobronchial tree. A patient with both pulmonary and extra-pulmonary TB is classified as a case of Pulmonary TB.
	**Extra-pulmonary TB** refers to any confirmed or clinically diagnosed case of TB involving organs other than the lungs
TB Outcome	**Cured** refers to a pulmonary TB patient with confirmed TB at the beginning of the treatment who was smear- or culture-negative in the last month of treatment and on at least one previous occasion.
	**Completed** refers to a TB patient who completed treatment without evidence of failure but with no record to show that sputum smear or culture results in the last month of treatment and on at least one previous occasion were negative, either because tests were not done or because results were unavailable.
	**Failed** refers to a TB patient whose sputum smear or culture is positive at month 5 or later during treatment.
	**Stopped** refers to cases where the health care team stops treatment of a TB patient.
	**Transferred out** refers to cases that are transferred to another treatment unit.
	**Defaulted** refers to a TB patient whose treatment was interrupted for two consecutive months or more.
	**Died** refers to a patient who dies for any reason during the course of treatment.
	**Missing** refers to a TB patient for whom no treatment outcome is assigned because information is missing and the treatment outcome is unknown to the reporting unit.
HIV status	**Positive** refers to a patient who has tested positive for HIV.
	**Negative** refers to a patient who has tested negative for HIV.
	**Inconclusive** refers to patient whose HIV testing results are inconclusive.
	**Refused** refers to a patient who refused to be tested for HIV.
	**Not done** refers to a patient who was not given a HIV-test.
	**Unknown** refers to a patient whose HIV status has not been recorded or recorded as unknown without further description.

Information about implementation quality was collected informally during quarterly PT meetings, field visits, and interviews with PTs and LHWs at intervention sites, in two companion qualitative studies reported separately.

Given the nature of the intervention and our pragmatic design, with use of routine Ministry of Health TB records for considering the scalability and sustainability of the intervention, blinding of participants and therefore recording of patient data and outcomes were not possible. Data abstractors were blinded to participant group.

### Sample size calculation

In the pilot study [15], a few HCs were found not to provide TB care and some clusters were lost because of staff shortages or failure to accrue eligible patients during the study period; therefore, although 109 HCs in four districts were eligible to participate, we estimated the sample size conservatively, using the approach outlined by Hemming et al. [22]. With an alpha of 0.05, power of 0.80, a baseline successful treatment completion rate of 0.80 at 1 year, intra-class correlation coefficient of 0.1 (based on the pilot study), and an estimated 100 clusters, we determined that a minimum of six patients per cluster was required to detect a clinically significant 0.10 increase in the proportion of patients with successful treatment completion.

### Statistical analysis

We calculated descriptive statistics for each district, including number of HCs, baseline characteristics, and TB outcomes across trial arms. Continuous outcomes were summarized as means and ranges, and categorical outcomes as frequencies and percentages. Intra-cluster correlation was estimated (by multilevel logistic regression) [23, 24] for the primary outcome and in the analysis of effectiveness for pulmonary and extra-pulmonary TB, respectively. All comparisons were two-tailed, with significance determined on the basis of alpha less than or equal to 0.05 level, and were conducted using R statistical software.

The primary analysis was conducted on an intention-to-treat basis and is reported according to the CONSORT guideline for pragmatic and cluster randomized trials [25, 26] (see CONSORT checklist, Additional file 2). After exclusion of HCs that accrued no eligible cases during the study period, one district was left with one stratum having a single cluster, which precluded the planned analysis with stratification by HC funding source. The primary outcome (TB treatment outcome) was first dichotomized into two categories: (1) those who were cured or who completed treatment and (2) those who did not complete the treatment. A generalized linear mixed model—with district as a fixed effect, HC as a random effect, and trial arm, age, sex, and TB type included to adjust for baseline imbalances between the trial arms—was then fitted to evaluate the impact of the intervention on proportion of treatment successes [27]. The 10 HCs that accrued no eligible patients and the 370 cases with missing outcome, HC, or demographic data were excluded from the primary analysis.

As the pilot study showed an interaction between intervention arm and TB type, a post hoc exploratory subgroup analysis was conducted to examine differences in outcomes across the various TB types. This analysis also used a generalized linear mixed model, with district as a fixed effect, HC as a random effect, and trial arm, age, and sex retained in the model. Given the small number of default cases (treatment interruption for at least two consecutive months), a planned secondary outcome analysis of proportion of default cases could not be conducted. In addition, a planned subgroup analysis of TB treatment outcome according to patient HIV status could not be conducted because some HIV status groups had no cases, making subgroup effect inestimable. While our trial protocol stated that our primary analysis would be conducted using a generalized linear mixed model, because of the non-collapsible nature of the logistic model, we wanted to confirm the primary outcome with a marginal model and therefore conducted a post hoc analysis using generalized estimating equations with an exchangeable correlation matrix.

Given the large number of cases with missing outcome data, we conducted further post hoc analysis to examine factors related to whether the treatment outcome was missing (with all outcomes other than “missing” grouped as outcome available). Univariate analysis stratified by district and outcome (available/missing) was conducted using chi-square for dichotomous variables and ANOVA for continuous variables. Multivariable analysis was conducted using a generalized linear mixed model, with sex, age, TB type, and trial arm included in the model and adjustment for nesting of cases within HCs within districts by nested random effects. Finally, we performed a best-case, worst-case sensitivity analysis [28], with all missing outcomes included as treatment success and then as treatment failures, to further assess the potential impact of missing outcomes.

### Protocol adaptations

The following adaptations from the published protocol were applied: planned stratification according to HC designation as a priority site for support and mentorship was not undertaken because of the small number of HCs designated as non-priority sites at the time of randomization; cascade training period was extended by 2 to 3 weeks, to address PT and LHW absences and delays in distribution of the training manual; digitization period was extended by 6 to 8 weeks beyond initial expectations to address high proportion of missing data thought to be due to delays in submission of TB treatment cards to registration sites; identifying data were not digitized for a few records at one non-intervention site (prevented by the HC’s management); loss of HCs that accrued no eligible patients during the study period prevented analysis by stratification by HC funding; and planned secondary outcome analysis of proportion of default cases and subgroup analysis of successes among cases with HIV co-infection were not completed because of insufficient data. We do not believe these adaptations would have had any important impact on our findings.

## Results

### Baseline characteristics and study flow

**Fig. 1:**
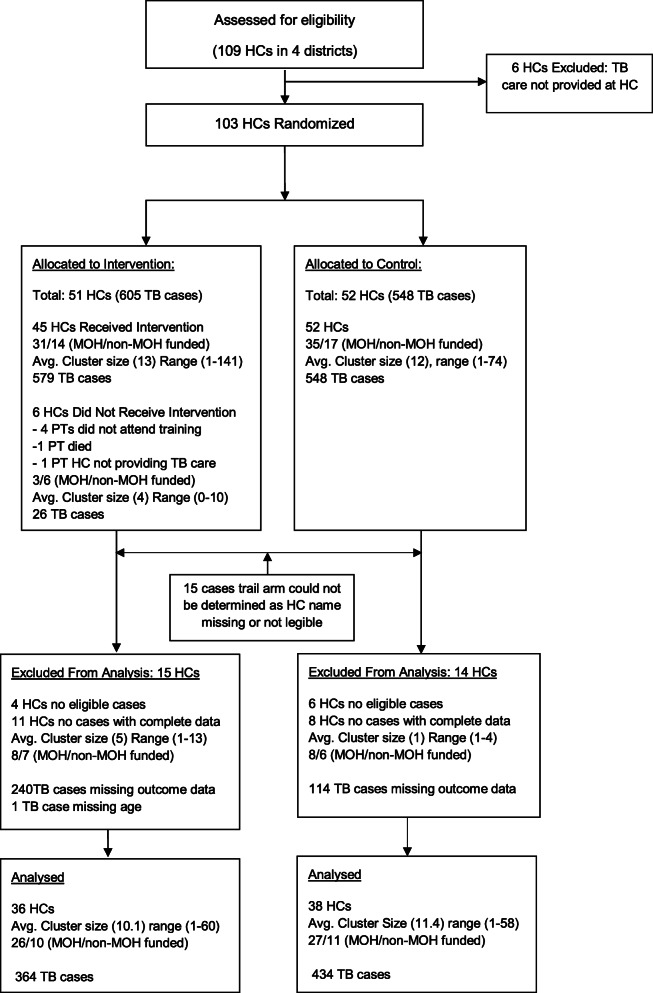
Details of flow of clusters and individuals through trial. HC health centre, MOH Ministry of Health, PT peer trainer, TB tuberculosis

Baseline characteristics for all cases and for cases included in the analysis are shown in Table 3. A total of 51 HCs were randomized to the intervention arm and 52 HCs to the control arm. Four HCs in the intervention arm and six in the control arm had no eligible patients during the study period, and 371 of 1169 patients had missing outcome data (241 in intervention arm, 114 in control arm), HC data (15 cases where name of treating HC was not recorded or not visible in the TB register), or demographic data (one case in intervention arm with age missing), which left 798 patients for analysis (Fig. 1). TB outcomes are shown in Table 4.

**Table 3:** Baseline characteristics for all data and complete data by trial arm

	Intervention (all data)	Control (all data)	Intervention (complete data)	Control (complete data)
**District level**				
Health centers (#/%)				
District 1	7/14.9	6/13	7/19.4	6/15.8
District 2	9/19.1	10/21.7	8/22	10/26.3
District 3	21/44.7	20/43.5	11/30.5	12/31.6
District 4	10/21.3	10/21.7	10/27.8	10/26.3
Cluster size (mean/range)				
District 1	7.1/1–12	24.5/1–71	7.1/1–10	23/1–58
District 2	13.3/1–85	7.9/1–21	13.6/1–60	7.1/1–19
District 3	13.6/1–141	6/1–29	5.8/1–32	3.8/1–10
District 4	15/1–64	15/6–74	14.1/1–61	17.9/6–65
Health centre funding (MOH/non-MOH)				
District 1	6/1	5/1	6/1	5/1
District 2	6/3	7/3	6/2	7/3
District 3	13/8	10/10	7/4	7/5
District 4	7/3	8/2	7/3	8/2
**Cluster level:**				
# of health centres	47^a^	46^a^	36	38
Cluster size (mean/range)	13/1–141	12/1–74	10.1/1–60	11.4/1–58
Health centre funding (MOH/non-MOH	32/15	30/16	26/10	27/11
**Patient level:**				
# of patients	605	548	364	434
Age in years (mean/range)	35.4/0–94	36.3/0–94	37.3/0–94	37.2/0–94
Women (#/%)	273/45.1	227/41.4	174/47.8	187/43.1
Pulmonary TB cases (#/%)	457/75.5	455/83	288/79	369/85
HIV status (#/%)				
Positive Negative Inconclusive Not done Unknown	310 (51.24) 285 (47.11) 1 (0.17) 0 (0) 9 (1.49)	284 (51.82) 254 (46.35) 0 (0) 1 (0.18) 9 (1.64)	200 (54.95) 164 (45.05) 0 (0) 0 (0) 0 (0)	225 (51.84) 201 (46.31) 0 (0) 1 (0.23) 7 (1.61)

**Table 4:** TB treatment outcomes by trial arm

Outcome	Intervention (***n*** = 605)	Control (***n*** = 548)
Cured	172 (28.43)	233 (42.52)
Completed	125 (20.66)	115 (20.99)
Failed	6 (0.99)	12 (2.19)
Stopped	1 (0.17)	0 (0)
Transferred out	7 (1.16)	7 (1.28)
Defaulted	4 (0.67)	6 (1.09)
Died	49 (8.10)	61 (11.13)
Missing	241 (39.83)	114 (20.80)

### Factors related to missing outcome data

Given the high proportion of missing outcome data, a post hoc analysis was conducted to examine factors related to whether or not the treatment outcome was missing. Univariate analysis employed chi-square for dichotomous (gender, TB type, randomization group) and ANOVA for continuous (age) variables stratified by district and outcome (outcome available/missing). A general linear mixed model was used to examine factors related to missing outcomes, with nested random effects used to adjust for the nesting of cases within HCs within districts. Table 5 shows the results of the univariate analysis and Table 6 the results of the regression analysis. The unadjusted analysis showed an association between randomization group and missing outcome, as well as for age and TB type, and missing outcome. However, in the model adjusted for age and TB type, the association of randomization group and missing outcome was no longer present (*p* = 0.30), implying that these factors were the important confounders. Age and TB type were therefore included in the primary analysis model, along with sex, which was pre-specified for inclusion on theoretical grounds.

**Table 5:** Results of univariate analysis of variables related to missing outcome data

	Missing total	Available total	Missing district 1	Available district 1	Missing district 2	Available district 2	Missing district 3	Available district 3	Missing district 4	Available district 4	*p*
*n*	354	799	9	188	19	180	301	110	25	321	
Sex = female (%)	138 (39.0)	362 (45.3)	3 (33.3)	88 (46.8)	9 (47.4)	78 (43.3)	116 (38.5)	47 (42.7)	10 (40.0)	149 (46.4)	0.59^1^
Age (mean (SD))	33.2 (16.8)	37.2 (16.7)	32.6 (14.7)	39.8 (16.2)	40.6 (18.2)	37.1 (17.6)	33.0 (17.2)	39.1 (19.0)	30.4 (9.5)	35.2 (15.5)	< 0.01^2^
TB type = extra-pulmonary (%)	100 (28.2)	141 (17.6)	2 (22.2)	31 (16.5)	8 (42.1)	41 (22.8)	85 (28.2)	16 (14.5)	5 (20.0)	53 (16.5)	< 0.01^1^
Trial arm = control (%)	114 (32.2)	434 (54.3)	9 (100.0)	138 (73.4)	8 (42.1)	71 (39.4)	80 (26.6)	46 (41.8)	17 (68.0)	179 (55.8)	< 0.01^1^

^1^Chi-square stratified by district and outcome

^2^Anova stratified by district and outcome

**Table 6:** Results of logistic regression analysis of variables related to missing outcome data

	Coefficient estimate	OR	95% CI	*p* value
*N* cases = 1153				
Sex = female	− 0.21	0.81	0.46–1.17	0.25
Age	− 0.01	0.99	0.98–1.00	0.01
TB type = extra-pulmonary	0.58	1.78	1.35–2.20	0.01
Randomized = control	− 0.19	0.82	0.46–1.19	0.30

### Primary outcome

Among the 1153 patients with HC and demographic data, 297/605 (49%) in the intervention arm and 348/548 (64%) in the control arm had successful treatment. Although crude proportions favoured the control arm, this effect is reversed in the adjusted analysis although it does not achieve statistical significance, likely because of associations with other variables included in the adjusted model. A small, non-significant improvement in successful treatment rate was found for the intervention arm, relative to the control arm (unadjusted odds ratio 1.16 [95% confidence interval (CI) 0.75–1.88]; adjusted odds ratio 1.35 [95% CI 0.93–1.98]) (Table 7). Analysis of the primary outcome using a generalized estimating equation with exchangeable correlation matrix yielded similar results (adjusted odds ratio 1.36 [95% CI 0.96–1.93]). The intra-cluster correlation coefficient (ICC) for the primary outcome was 0.04. The best-case, worst-case sensitivity analysis (with all missing outcomes included as treatment successes and as treatment failures) found relative successful treatment rates in the intervention, and control arms were essentially unchanged (best-case scenario, adjusted odds ratio 1.44 [95% CI 0.10–2.09]; worst-case scenario, adjusted odds ratio 1.04 [95% CI 0.74–1.46]). In addition, the statistical significance of age differed by scenario, becoming non-significant for the worst-case scenario).

**Table 7:** Logistic regression results of primary analysis of effectiveness of intervention in improving proportion of cases successfully treated

Variables	Unadjusted		Adjusted	
	OR	95% CI	OR	95% CI
*N* cases = 798, ICC = 0.035				
Randomization arm–intervention vs. control	1.16	0.75–1.88	1.35	0.93–1.98
District 1 vs 2	2.35	1.37–4.13	2.63	1.50–4.72
District 1 vs 3	2.56	1.41–4.76	2.94	1.58–5.53
District 1 vs 4	1.35	0.81–2.30	1.56	0.92–2.70

### Secondary outcome

Planned secondary outcome analysis of the proportion of default cases could not be conducted because of the small number of such cases, four (0.67%) and six (1.19%) in the intervention and control arms, respectively.

### Subgroup analysis

TB treatment success rates were similar among HIV-positive and HIV-negative cases in both the intervention and the control arms (Table 8): 25.29 and 23.80%, respectively, in the intervention arm, and 30.84 and 31.57%, respectively, in the control arm (percentages based on total number of TB cases, including those with missing TB outcome). However, the planned subgroup analysis of TB treatment success according to patients’ HIV status could not be conducted because some HIV status groups had no cases.

**Table 8:** TB treatment outcome by HIV status

	Intervention (***n*** = 605)			Control (***n*** = 548)		
	HIV status			HIV status		
**TB outcome**	HIV-positive	HIV-negative	HIV-status other^a^	HIV-positive	HIV-negative	HIV status other^a^
Cured Completed Treatment success	71 (11.74) 82 (13.56) 153 (25.29)	101 (16.69) 43 (7.11) 144 (23.80)	0 (0) 0 (0) 0 (0)	100 (18.25) 69 (12.59) 169 (30.84)	130 (23.72) 43 (7.85) 173 (31.57)	3 (0.55) 4 (0.73) 7 (1.28)
Failed Stopped Transferred out Defaulted Died Treatment unsuccessful	4 (0.66) 1 (0.17) 5 (0.83) 2 (0.33) 35 (5.79) 47 (7.77)	2 (0.33) 0 (0) 2 (0.33) 3 (0.50) 14 (2.31) 21 (3.47)	0 (0) 0 (0) 0 (0) 0 (0) 0 (0) 0 (0)	6 (1.09) 0 (0) 6 (1.09) 2 (0.36) 42 (7.66) 56 (10.22)	6 (1.09) 0 (0) 1 (0.18) 3 (0.55) 18 (3.28) 28 (5.11)	0 (0) 0 (0) 0 (0) 0 (0) 1 (0.18) 1 (0.18)
Missing	110 (18.18)	120 (19.83)	10 (1.65)	59 (10.77)	52 (9.49)	3 (0.55)

^a^Other includes inconclusive, not done, and outcome missing

Given the significant model effect found for TB type (pulmonary vs. extra-pulmonary TB) and findings in our pilot study of a significant effect of TB type in the control arm, with reduced treatment completion rates among extra-pulmonary TB cases, we conducted a post hoc analysis of the effect of the intervention according to TB type (Table 9). The ICCs for pulmonary and extra-pulmonary TB were 0.03 and 0, respectively. Although the odds of treatment success were somewhat higher for pulmonary TB (adjusted odds ratio 1.45 [95% CI 0.95–2.25]) than for extra-pulmonary TB (adjusted odds ratio 0.91 [95% CI 0.29–2.62]), no significant effect of the intervention on treatment success was found for either type.

**Table 9:** Logistic regression results of TB type sub-group analysis of effectiveness of intervention in improving proportion of cases successfully treated

Variables	Unadjusted		Adjusted	
	OR	95% CI	OR	95% CI
Pulmonary TB ( *N* = 657, ICC = 0.03)				
Trial arm (intervention vs control)	1.33	0.82–2.27	1.45	0.95–2.25
District				
District 1 vs 2	0.41	0.21–0.78	0.35	0.18–0.68
District 1 vs 3	0.38	0.18–0.74	0.33	0.16–0.67
District 1 vs 4	0.65	0.35–1.17	0.58	0.31–1.06
Extra-pulmonary TB (*N* = 141, ICC = 0)				
Trial arm (intervention vs control)	0.87	0.36–1.83	0.91	0.29–2.62
District				
District 1 vs 2	0.56	0.14–1.59	0.41	0.06–1.65
District 1 vs 3	0.38	0.10–1.38	0.31	0.05–1.80
District 1 vs 4	1.11	0.35–3.45	0.99	0.22–5.33

### Implementation outcomes

Implementation outcomes are summarized in Table 10. Forty-eight TB-focus LHWs were trained as PTs, with four intervention sites, not represented at training as their TB-focus LHWs were away from work and/or attending other trainings. Upon completion of training, one PT reported that TB care was no longer provided at their site. In addition, one PT died a few weeks after completing training. As a result, six sites did not have the opportunity for cascade training, and an additional four sites had no LHWs with training completed at the end of the study; however, all 52 intervention sites were included in the analysis, as randomized. In total, 152 LHWs were reported to have completed the cascade training by the end of the initial training period (not including LHWs at one site where the PT initially reported training completion, with later acknowledgement that training was incomplete). The proportion of LHWs who received cascade training ranged from 0 to 100% across sites; lack of training stipends was the primary reason for LHWs declining to participate. Several PTs reported provision of training to LHWs who initially declined training and/or were transferred into the HC after the initial training period, such that a total of 169 LHWs had received the cascade training by the end of the study. Of these, 157 remained at intervention sites at the end of the study; eight transferred out (two to other intervention sites), one left for a new job, two left to go back to school, and one died. Final reports were not available for seven HCs whose PTs did not attend the final meeting and could not be reached by phone to confirm final numbers. LHWs who transferred to non-intervention sites were asked not to share their learning with staff at their new site and to leave their point-of-care tools at their original intervention site (to prevent sharing with non-intervention site staff). In addition, a few LHWs interviewed in the process evaluation (unpublished data) and leadership sub-study reported elsewhere [29] reported that their training was condensed or incomplete. All sites where cascade training was conducted reported ongoing use of their training and point-of-care tool in provision of care to the end of the study.

**Table 10:** Implementation outcomes

Implementation outcomes	Implementation outcome results
HCs receiving cascade training	42 of 51 sites completed cascade training
PTs trained	48 of 51 invited completed PT training
LHWs completing cascade training	152 LHWs completed cascade training during initial training period 169^a^ LHWs completed cascade training by study end
Training adherence	Adherence to training content and process including frequency and duration of training varied significantly
HCs using intervention at study end	All HCs with trained LHWs reported continued routine use of the intervention at study end
Trained LHWs at HC at study end	157 trained LHWs remaining on site • 8 transferred out (two to other intervention sites) • 2 left to return to school • 1 left for a new job • 1 died

^a^End of study numbers do not include reports from seven PTs who could not be reached to confirm final numbers

## Discussion

Consistent with the findings of our pilot study, although TB treatment success rates were higher in the intervention arm after adjustment for baseline imbalances, the difference was not statistically significant. In contrast to the pilot study, the intervention had no significant effect on treatment success for either TB type (pulmonary or extra-pulmonary). Age and TB type were significantly related to missing outcomes in the adjusted model, whereas randomization group was not. We also found high variability in implementation quality, which highlights important challenges to both scale-up and sustainability.

Although the loss of some clusters and the high proportion of missing outcomes may have contributed to these findings, we adjusted for the impact of missing outcomes in the primary analysis by including factors related to missing outcome, and the lower-than-anticipated ICC would have increased power to detect an effect. In addition, low implementation quality both within and across districts was likely an important contributing factor. Although many PTs achieved high levels of participation and high-quality implementation at their sites, a substantial proportion of LHWs opted not to participate in the cascade training, which led to low levels of reach, adoption, and implementation at many intervention sites [18]. The principal reason for refusal to participate was lack of training stipends. In addition, reports from LHWs in a companion study evaluating the impact of PT leadership style on uptake of the intervention [29] noted a few cases where PTs did not provide the complete training package. Implementation quality at some sites was low, despite additional (if limited) supports and mentorship provided through contact with the study team and Dignitas trainers. The quality of implementation might be worse under routine programmatic conditions, where intense program support would not be feasible because of resource constraints in the Malawi health care system.

The low implementation quality contrasts with our pilot study, where LHW participation in cascade training was high, despite lack of stipends. Although training stipends were common practice at the time of the pilot study, we chose not to provide them, to evaluate implementation of the intervention as pragmatically as possible, given that training stipends could not feasibly be provided with national scaling-up. By the time of the current study, training stipends were no longer allowed by Ministry of Health policy, with training refusals occurring among other health worker cadres as a result of this change.

Additional potential contributing factors to the absence of an intervention effect on TB treatment outcomes include PT selection and training. TB-focus LHWs were selected as PTs because of their additional TB-specific training and role. However, reports from LHW participants in the concurrent qualitative studies indicate that the PTs’ commitment and leadership approach played an important role in uptake of the intervention. As such, additional selection criteria and/or leadership training may be important to improve uptake and sustainability of the intervention.

Our pilot study showed an interaction between TB type and study arm, whereby reduced treatment completion rates for extra-pulmonary TB were found only in the control arm [15]. This effect, hypothesized to result from increased patient understanding of TB as a result of the intervention, was not observed in the current study. Rather, the subgroup analysis showed higher TB completion rates for pulmonary TB than for extra-pulmonary TB, although the difference did not achieve statistical significance. This difference may be related to factors such as the relatively small proportion of extra-pulmonary TB cases in both studies, possibly resulting in a spurious association, or the relatively better education provided for patients with pulmonary TB in the current study.

Previous systematic reviews have shown LHWs to be both effective [4, 5] and cost-effective [30] in improving outpatient TB treatment outcomes, but relatively few studies have evaluated interventions designed to improve TB care provided by LHWs and thus to improve TB outcomes. Okeyo et al. [31] developed and evaluated a 17-page booklet to reinforce LHW knowledge and facilitate patient counselling. LHWs’ TB-related knowledge and self-reported confidence increased, but the impact on patients’ TB outcomes was not assessed.

Several studies have assessed interventions implemented using peer-led training and/or tools to support LHWs’ work in areas other than TB, but they did not assess the impact of the intervention on patient outcomes. Siribie et al. [32] trained LHWs in four LMICs to manage malaria, with two countries using a cascade peer training approach in which LHW supervisors were trained as PTs. Most LHWs successfully completed the training and achieved high levels of performance, in patient assessment, diagnosis, and treatment criteria. Yu et al. [33] trained LHW supervisors as PTs to provide cascade training to junior LHWs for a water, sanitation, and hygiene program in Haiti. They found a significant increases in PT knowledge and observed ability to provide cascade training; however, knowledge gains were not sustained, with no difference between participants and non-participants 1 year after the intervention; this result suggests the need for refresher training. Gualy et al. [34] developed and evaluated a pictorial guide to aid community health workers in Honduras to recognize and refer patients with surgical disease. The study, which involved a mixed group of community health workers both formal and informal, examined the effectiveness of the guide either on its own or combined with a curriculum led by medical students; knowledge improved significantly in both scenarios, but effect on patient outcomes was not assessed.

In our pilot study, fear of knowledge testing was thought to be a barrier to LHWs’ participation in the training and intervention implementation; as such, we did not formally assess change in knowledge in the current study. However, LHWs in the current study reported increased TB knowledge and increased confidence in their work with TB patients.

As in our pilot study, the intervention had no significant effect on TB treatment outcomes. However, implementation on a larger scale and under different policy conditions in the current study has helped to highlight important challenges to both scale-up and sustainability of the implementation strategy, as well as challenges to use of this approach for LHW training in other areas of care. Given the recognized need to address LHW training needs on a cost-efficient and ongoing basis, and given that training stipends are not feasible or sustainable, other factors to promote the implementation of interventions that employ on-site peer-led training must be explored and evaluated. Participation in training and implementation may improve under regular programmatic conditions, if it is made a staff requirement; other options to increase participation for future evaluation include engaging local opinion leaders or champions as PTs, training supervisors to support implementation, and providing leadership training as part of PT training.

## Strengths and limitations

The strengths of this study included pragmatic design and concurrent process evaluation. Implementation with limited support from the study team allowed for evaluation of effectiveness under conditions feasible for scale-up, increasing the generalizability of our findings. Our concurrent process evaluation (reported separately) revealed important challenges and opportunities for the scaling-up and sustainability of the intervention and the implementation strategy reported here and for use of this approach to address training needs in other areas of care provided principally by LHWs.

The study limitations included the high proportion of cases missing outcome data, lack of blinding, and use of TB registry data. Although loss of clusters that accrued no eligible cases occurred in both the pilot and current studies, outcome data were largely complete in the pilot study. Several factors may have contributed to the completeness of outcome data in the pilot study. Outcome data were collected more frequently than in the current study, which may have encouraged completion of records. A longer period of support and training of clinical staff (provided by Dignitas) may have improved record keeping in the pilot district. Finally, outcome data were obtained from HC treatment cards in the pilot study, whereas the current study used TB register data, which relied on return of treatment cards to registration sites. Extension of the data collection period should have been sufficient to address delayed return of treatment cards, but data were also missing for some patients treated at registration sites, where there was no such delay.

Loss of clusters that accrued no eligible cases and the high proportion of missing outcomes may have reduced the power to detect an intervention effect. However, the lower-than-anticipated ICC may have mitigated the loss of power, and as CIs were narrow precision of the estimate does not appear to have been substantially affected. Despite the substantial imbalance in proportion of cases with missing outcomes between the intervention and control arms, missing outcome was found not to be related to randomization group in the model once adjusted for identified sources of bias. In considering how best to address missing data, multiple imputation was considered but deemed inappropriate because of the large numbers of clusters having a single case with outcome data (i.e., insufficient information in the clusters to allow for proper imputation). Because all but one case had complete independent variable data, and because prognostic variables significantly related to missingness were identified and included, complete case analysis was appropriate [28, 35, 36]. The non-significance of effect of trial arm in the best-case, worst-case sensitivity analysis further supports the assumption that outcomes were missing at random and therefore the appropriateness of using complete case analysis with inclusion of variables related to mechanism [27]. The planned subgroup analysis of treatment success according to HIV status could not be conducted because some HIV status groups had no cases, making subgroup effect inestimable.

As noted by our Malawi TB program partners, collection of outcome data several months beyond when these outcomes should have been recorded is an important finding in itself and suggests the need for further work to facilitate appropriate and timely documentation and thus ensure accuracy of Ministry records. In addition, blinding to intervention was not possible because of the nature of the intervention and the use of Ministry of Health TB records. However, given the variability in implementation quality, lack of blinding is unlikely to have had a substantial effect on our findings. It is possible that some cases with outcomes of “cure” or “treatment complete” may have been misclassified. Although we found no such discrepancies in our review of the data, some patients who qualified for the outcome of “cure” may have been recorded as “treatment complete” because not all sputum results were not recorded. However, these two outcomes were grouped into a single category for treatment success, so this would not have affected our analysis or findings.

## Conclusions

We found no significant effect of the intervention on TB treatment outcomes. The high variability in implementation quality highlights important challenges to both scale-up and sustainability. Future work to explore and evaluate approaches to addressing these challenges is needed before the current program can be scaled-up and the approach used to address LHW training and supervision needs in other areas of care may be considered.

## Supplementary Information

**Additional file 1.** TIDieR checklist

**Additional file 2.** CONSORT checklist

## Data Availability

The datasets used and/or analysed during the current study are available from the corresponding author on reasonable request.

## Abbreviations

HC: Health centre
ICC: Intra-cluster correlation coefficient
KT: Knowledge translation
LHW: Lay health worker
LMICs: Low- and middle-income countries
NGO: Non-governmental organization
PT: Peer trainer
RA: Research assistant
TB: Tuberculosis
